# DFT Studies of Selected Epoxies with Mesogenic Units–Impact of Molecular Structure on Electro-Optical Response

**DOI:** 10.3390/ijms22073424

**Published:** 2021-03-26

**Authors:** Magdalena Włodarska, Beata Mossety-Leszczak

**Affiliations:** 1 Institute of Physics, Faculty of Technical Physics, Information Technology and Applied Mathematics, Lodz University of Technology, Wólczańska 219, 90-924 Łódź, Poland; 2 Department of Industrial and Materials Chemistry, Faculty of Chemistry, Rzeszow University of Technology, al. Powstańców Warszawy 6, 35-959 Rzeszów, Poland; mossety@prz.edu.pl

**Keywords:** liquid crystal, theoretical calculations, dipole moment, epoxy materials

## Abstract

Theoretical studies of molecular structure and electric charge distribution were carried out for three epoxy compounds with different mesogenic cores. The compounds exhibit a nematic phase and form polymer networks that are potential bases for various composites. Results were compared to analogous materials with non-polar chains. A customized process involving geometry optimization of a series of conformations was employed to greatly increase likelihood of reaching global energy minimum for each molecule. All computations used Density Functional Theory (DFT) electron correlation model with the B3LYP hybrid functional. Molecular structure calculations yielded several parameters, including the magnitude and direction of the dipole moment, polarizability (α), first hyperpolarizability (β), and highest-occupied/lowest-unoccupied molecular orbital (HOMO-LUMO) energies. These parameters can help predict electronic properties of the nematic phase and the polymer network and assess their predisposition for application in electrooptical devices. In particular, the magnitude and direction of the dipole moment determine molecular alignment of liquid crystal phases in electric field, which enables controlling molecular order also in cured networks. Theoretical results were supplemented with observations of the nematics and their behavior in electric field. It was demonstrated for the studied compounds that a change in aliphatic chain polarity helps preserve and reinforce perpendicular alignment of molecules induced by electric field.

## 1. Introduction

Liquid crystal (LC) materials have found many applications in electrooptical devices [1,2]. In particular, they are still widely used in traditional LC displays [3,4]. In addition to standard LC properties, liquid crystals epoxies (LCE) also possess the ability to form liquid crystal polymer networks (LCPN) with well-defined structure and controllable molecular alignment [5,6]. The materials studied in this work were successfully used as precursors for polymer matrices with unidirectional alignment of the monomers [5,6]. Such properties seem very desirable from the perspective of their potential applications in organic electronics and nonlinear optical devices. LCPN can also be used to create advanced organic/inorganic hybrid composites with desired electro-optical properties, being also mechanically and chemically resistant. For example, modifying a polymer network with nanoparticles containing optically active functional groups (such as azo groups) provides a way to induce photochromic properties into a composite [7]. Theoretical methods can be used to assess the potential for non-linear response in an LCPN.

All the investigated compounds have a nematic phase at elevated temperatures. The main parameter determining the molecular order of a nematic in an electric field is the dielectric anisotropy Δ*ε* defined as the difference between two values of the dielectric constant—*ε*∥ and *ε*_⊥_—measured, respectively, in the direction parallel and perpendicular to the nematic director *n* (along and across molecules). The corresponding contribution to free energy of a nematic in an electric field *E* is [8,9,10]:

(1) $$E_{E}=-\frac{\varepsilon_{⊥}E^{2}}{8\pi }-\frac{\Delta \varepsilon {\left(n\cdot E\right)}^{2}}{8\pi }$$

Δ*ε* may be positive or negative [8,9,10]. When a nematic material with positive dielectric anisotropy is placed in an electric field exceeding a certain threshold, its molecules tend to assume a homeotropic alignment (parallel to the direction of the electric field and perpendicular to the cell surface). If the dielectric anisotropy is negative, the dominant orientation of the molecules is perpendicular to the direction of the electric field [9,10,11]. A correlation between the dielectric anisotropy and the molecular structure was described in detail by the theory of Maier and Meier [9,10,12]. Specifically, the sign of the dielectric anisotropy can be linked to the orientation of the total dipole moment with respect to the principal molecular axis: a large transverse component of the dipole moment is indicative of negative anisotropy [9,10]. Thus, knowing the value of that parameter enables predicting the behavior of a nematic material in an electric field. This is the basis of display control in traditional LC displays.

In the case of the investigated LCEs, the molecular alignment can also be controlled during curing, and partially preserved in the cured structure. So far, successful attempts to create such ordered networks were made using an external magnetic field during the reaction [5,6]. It is worth noting that the contribution to free energy of a nematic in a magnetic field *H* has a similar form:

(2) $$E_{H}=-\frac{1}{2}\chi_{⊥}H^{2}-\frac{1}{2}\Delta \chi {\left(n\cdot H\right)}^{2}$$

where *χ*_⊥_ is magnetic susceptibility component perpendicular to the director. The anisotropy of magnetic susceptibility Δ*χ* is positive in most nematics, which favors molecular alignment along the lines of the magnetic field. On the contrary, the dielectric anisotropy may have either sign, depending on the material [8,9,10]. Theoretical calculations of the dipole moment and its direction can be used to predict LC behavior in an electric field (also during curing). They can also yield other parameters, such as polarizability α and hyperpolarizability β [13], which are significant from the perspective of applying liquid crystal epoxy matrices in electrooptical devices. The hyperpolarizability component along the active layer can be connected with macroscopic second-order susceptibility tensor. As an example, in a study of some other liquid crystal organic materials with rigid, bent mesogens (aiming at their applications in nonlinear optics) the authors described the characteristic parameters *D* and *d* in terms of just two hyperpolarizability components [1]:

*D* = *Nf*^3^ < cos *φ*>*β_zxx_ d* = *Nf*^3^ < cos^3^*φ*>*β_zzz_* (3)

where *N* is the density of molecules in the mesophase, *f*—local field factor, and *φ* is the angle between the *z* axis and the spontaneous polarization direction. In that case, the obtained values of *d* = 48 pmV^−1^ and *D* = 140 pmV^−1^ were much larger than in a traditional crystal, KDP (KH_2_PO_4_; d_14_ = 0.5 pmV^−1^, d_36_ = 0.46 pmV^−1^ [1]). Large optical nonlinearities were reported in organic crystals based on nitrobenzene, aniline, or chalcone derivatives (with d_14_ = 8.82 pmV^−1^) [14,15]. Optoelectronic properties are also governed by intermolecular charge transfer (ICT), light transmittance, and energy gap width. ICT originates mainly from donor-to-acceptor moieties via *π*-conjugated bridges. Substitution of electron acceptors and donors on any of the phenyl rings greatly influences the non-centrosymmetric packing structures required for second harmonic generation [14]. Frontier molecular orbital (FMO) computations enable determination of the energy gap between HOMO (electron donor) and LUMO (electron acceptor). In design and synthesis of new polymers scientists have been working to lower the FMO energy levels and to obtain a wide range of new electron-withdrawing and electron-donating conjugated building blocks. FMO and molecular self-assembly property are considered the key factors determining the charge carrier transport and they condition potential applications of organic materials in electronics [16].

The present paper compares results of DFT calculations for three epoxy compounds with long aliphatic chains and two of their vinyl analogs. The computed values of the magnitude and direction of the dipole moment enable prediction of the molecular alignment in an electric field in the nematic phase and during the curing reaction, whereas higher-order parameters, such as polarizability and hyperpolarizability, help to assess the behavior of molecules in an electric field in the cured products. Experimental work conducted in parallel for the same materials gave quite promising results regarding their use as basis for molecularly oriented polymer networks [5,6]. In an earlier work, ab initio calculations were carried out at Hartree–Fock (HF) level of theory for two pairs of molecules with short aliphatic chains attached to a bi-aromatic mesogen, in vacuum and in solution [17]. That former study demonstrated a change in the sign of the dielectric anisotropy after introducing epoxy groups in the pair of molecules with aromatic rings linked by carboxyl groups [17]. DFT results reported in the present paper hint, in most cases, at perpendicular alignment of molecules with respect to the electric field, which has been confirmed experimentally in the nematic phase. Such behavior makes it possible to use an electric field during curing to obtain ordered LCPN. These novel observations complement earlier experimental studies on the same materials which confirmed the possibility to affect the alignment of molecules in created polymer matrices by applying an external magnetic field during the reaction [5,6] Nevertheless, the behavior of these materials in a static electric field remains a subject of study.

## 2. Results and Discussion

### 2.1. Choosing the Best Conformation—DFT Optimization

**Figure 1:**
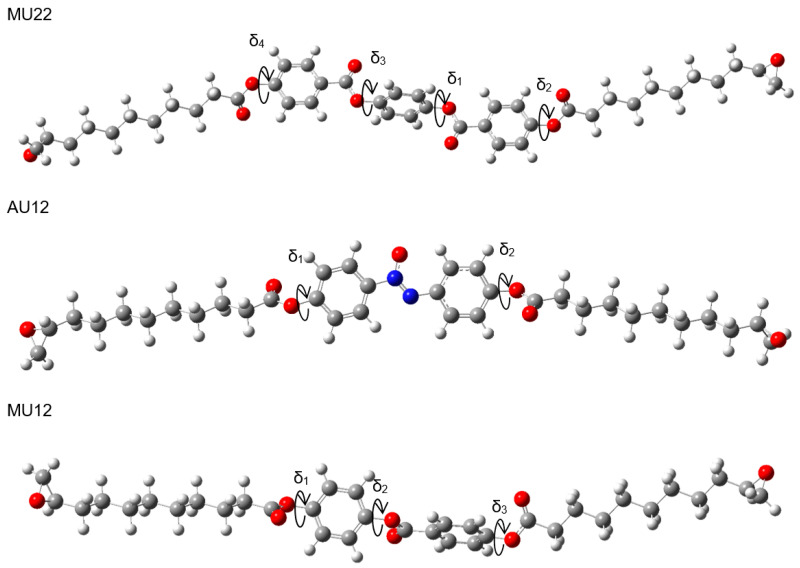
Spatial structure of the three epoxy compounds. Key dihedral angles defining the conformation (δ*_i_*) are indicated by circular arrows.

Geometry optimization of any but the smallest molecules is not a trivial process, because automated energy-minimization methods may stop at a stationary point which is only a local minimum, rather than the desired global minimum. The molecules studied in this work can be structurally divided into three segments: a central, rigid mesogen which is responsible for inducing the liquid crystal phase, and two identical, flexible, aliphatic chains symmetrically attached to the mesogen. In this way, the optimization process can be improved by pre-optimizing the segments and then exploring different possible arrangements of the segments. One rigid core (containing two rings and three ester groups) is shared by molecules M12 and MU12, whereas another central segment (made of three rings and four ester groups) is common to molecules M22 and MU22. Thus, along with the mesogenic core of AU12, three central segments and two aliphatic chains were pre-optimized in the first step. We then performed geometry optimization procedure for complete structures. In our previous work studying other liquid crystal compounds with similar structures we noted that spatial conformation of such molecules is defined by dihedral angles between aromatic rings and ester groups within the central segment, which are not coplanar in optimized geometries [17]. Moreover, it is possible to construct several conformations by independently setting each angle to either +δ or −δ (twisting the bond clockwise or anti-clockwise). Geometry optimization of such structures would likely converge to different local minima of energy, due to the energy barrier between both orientations. This is important because ester and azoxy groups are polar, so conformational changes of the central segment may significantly affect its total dipole moment and, for instance, determine the electrical behavior of the material in liquid crystal and solid phase. We applied this reasoning to the studied compounds, considering a series of potential conformations which differ in the mutual orientation of polar groups and aromatic rings. The key dihedral angles are shown in Figure 1 for each of the epoxy molecules.

**Figure 2:**
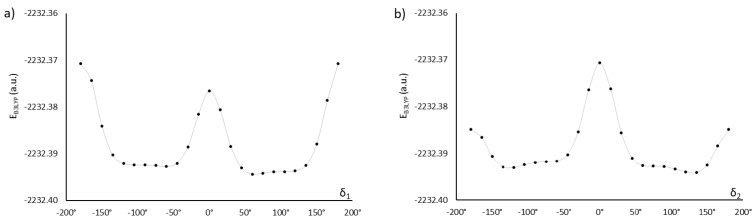

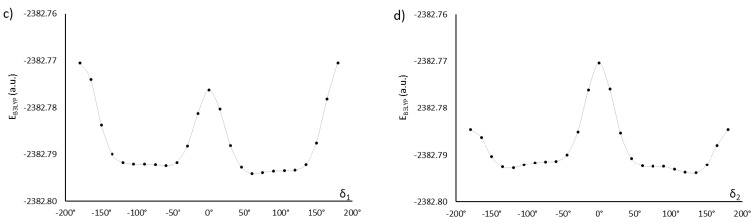
Energy changes in relation to the mutual orientation of phenyl rings and ester groups (described by dihedral angles δ_1_ and δ_2_) in monomers M22 (**a**,**b**) and MU22 (**c**,**d**). The solid lines are guides to the eye.

The monomers MU22 and M22 share the same mesogen, with key angles δ_1_, δ_2_, δ_3_, and δ_4_. Only two of them are independent, however, since we may expect symmetry in ground-state conformations of symmetric chemical structures. Thus, a conformation is fully defined by two angles (e.g., δ_1_ and δ_2_) and the symmetry, which determine the other two angles. Figure 2 shows how altering the angles δ_1_ (δ_3_) and δ_2_ (δ_4_) affects the potential energy of the molecules MU22 and M22. In both monomers the two symmetrical local minima (+δ and −δ) are visible, as well as the energy barriers between them. The minima for δ_2_ (δ_4_) located at about ±120° are better defined, whereas the minima for δ_1_ (δ_3_) at about ±75° are broad and flat, suggesting some degree of rotational freedom at those bonds.

Structures with central, mirror, and two rotational symmetries can be obtained by making δ_3_ equal to δ_1_, −δ_1_, δ_1_−180°, or 180°−δ_1_, followed by setting δ_2_ and δ_4_ to ±δ_2_ in accordance with the given symmetry. Combining four configurations of the inner ester groups (δ_1_, δ_3_) with two orientations of the outer ester groups that match the overall symmetry (δ_2_, δ_4_) yields eight distinct, potentially stable conformations of the molecule. These structures were finally optimized at DFT (B3LYP) level of theory—the values of DFT energy, as well as the magnitude and direction of the dipole moment, are presented in Table 1 for M22 and MU22, respectively. In all the studied conformations, the total dipole moment is nearly perpendicular to the principal axis of the molecule, which predicts planar alignment of molecules in the nematic phase under the influence of an electric field. Since the total dipole moment depends on mutual orientation of polar groups in the mesogen and in the chains, it may be small even for molecules with many polar groups. In M22, four conformations have mesogens with the total dipole moment close to zero, whereas the other four conformations have mesogens with larger dipole moments. We decided to consider one conformation with the lowest energy from each group (denoted in Table 1 by M22_α_ and M22_β_) in further computations, in order to assess differences between other electronic properties of more- and less-polar configurations. We also selected two analogous conformations of MU22 (based on the same mesogen configurations as M22_α_ and M22_β_, one being non-polar and the other one polar), denoted MU22_α_ and MU22_β_. For all selected conformations, indicated in Table 1 by highlighting, partial dipole moments of the central segment and the terminal chains are also presented. This helps to understand the influence of a given polar group on the total dipole moment.

The next investigated compound is AU12, which has a mesogen containing an azoxy group. A very similar mesogen terminated with methyl groups gives rise to p-azoxyanisole (PAA), one of the earliest-known nematic materials with negative dielectric anisotropy. That negative anisotropy was commonly attributed to the presence of a polar azoxy group in the mesogen, with dipole moment perpendicular to the molecular axis. Even though the first papers on PAA were published in the 1950s, it continues to attract research interest in both the experimental and theoretical field [18,19,20,21,22,23]. In comparison with PAA, the structure of AU12 contains additional carboxyl groups at both ends of the mesogen and aliphatic chains terminated with polar epoxy groups. Nevertheless, the earlier studies on PAA may be a point of reference for our work due to similarity of the mesogens.

**Figure 3:**
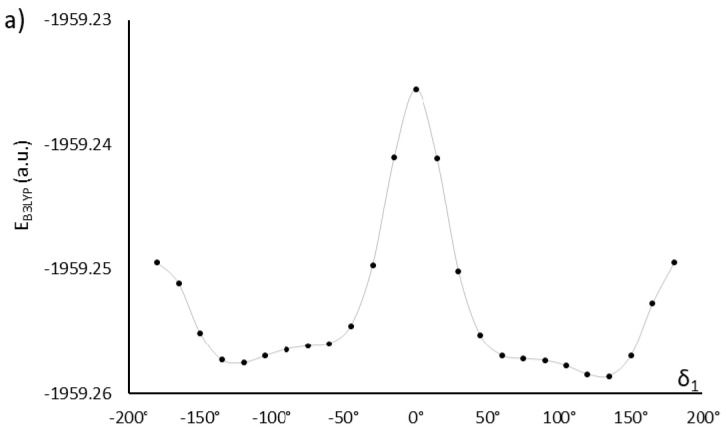

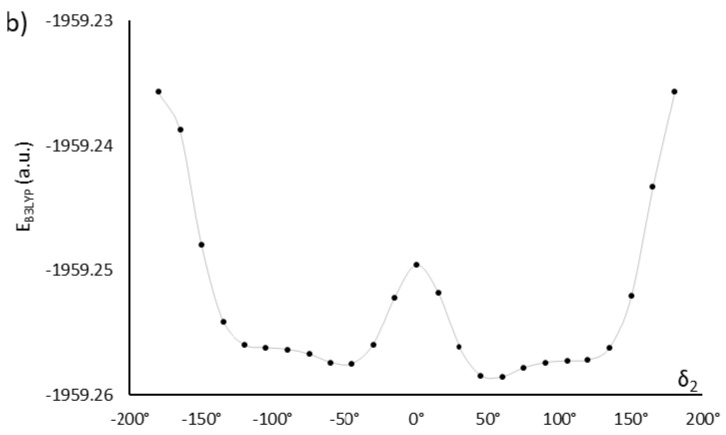
Energy changes in relation to the mutual orientation of phenyl rings and ester groups, described by dihedral angles δ_1_ (**a**) and δ_2_ (**b**) in AU12 monomer. The solid lines are guides to the eye.

Exhaustive optimization of molecular structure of AU12 follows the approach already discussed for MU22. In this case, there are only two key dihedral angles, because both phenyl rings in the mesogen are coplanar with the azoxy group. This is also a property of PAA [20,21,22,23] and added carboxyl groups do not disrupt that coplanarity. However, the carboxyl groups themselves are not coplanar with the phenyl rings; their orientation is given by dihedral angles δ_1_ and δ_2_ (Figure 1), with energy minima located near ±120° and ±60° (Figure 3).

The two possible choices for δ_1_ (the other two are mirror reflections) and four orientations of the other carboxyl group (δ_2_) give rise to eight distinct conformations. In result of geometry optimization, they converged to eight different stationary points indeed, with energy values and dipole moments shown in Table 2. For further analysis, we selected the conformation with the lowest energy and one conformation with low energy and a larger dipole moment of the mesogen—like in the case of the first pair of compounds. It is noteworthy that the dipole moment of AU12_β_ conformer is much larger than in AU12_α_. In this molecule, orientation of the polar epoxy chains (whose dipole moments are comparable with the mesogen) also has large impact on the value of total dipole moment (Table 2).

The analysis of the last pair of compounds (M12/MU12) is based on earlier considerations carried out for the same mesogen as part of a study on similar liquid crystal materials with shorter chains [17]. To identify all local minima of energy, we analogously modified the angles δ_1_, δ_2_, and δ_3_ (see Figure 1) to construct structures with different arrangement of intramolecular planes and performed final optimization of those structures. For both molecules, the dependency of B3LYP energy on the values of specific dihedral angles exhibits local minima located at about ±60° for δ_1_ and δ_2_, or about ±45° for δ_3_. All these minima are rather broad and flat; therefore, their corresponding bonds are expected to have some rotational freedom. Like before (MU22/MU12), eight candidate conformations were identified by setting δ_1_ to 60°, picking one of four orientations of the middle carboxyl group δ_2_ (±60° or ±120°), and setting δ_3_ to ±45°. All the candidates converged to eight different final structures in result of geometry optimization. Interestingly, the values of dihedral angles δ_i_ after optimization (δ_1_ ≈ 46°, δ_2_ ≈ 135°, δ_3_ ≈ 40°) differ noticeably from their initial values. This observation is consistent with flatness of the energy surface in vicinity of the minimum and further supports the expectation of certain rotational flexibility of these bonds. Table 3 displays B3LYP energy values for all the optimized conformers of both molecules. Unlike the case of MU22/M22, the lowest energy does not correlate here with low total dipole moment. This can be attributed to asymmetric structure of the mesogen, which remains polar regardless of conformation.

Summarizing the above discussion: two conformations of each molecule (with small and large dipole moments of the mesogens) were selected for further analysis. Substantial differences were noticed in the magnitude of the dipole moment, depending on mutual orientation of polar groups in the conformer; this includes both the arrangement of polar groups inside a mesogen and the orientation of terminal chains with respect to the mesogen. Therefore, knowing only the magnitude of the total dipole moment does not provide enough information about molecules containing several polar groups, because the electric field interacts with all the individual polar groups. It can be noted that polar groups in the aliphatic chains did not affect the overall conformation and the dipole moment of the mesogen, since very similar results were obtained from optimization of analogous epoxy and vinyl molecules. It is also meaningful that *Θ*—the angle between the direction of the total dipole moment and the principal molecular axis—is between 63° and 90° in most structures, so the transverse component of the dipole moment dominates. The only exception is M12, where *Θ* is 38° and 51° in the two selected conformers, which is not a clear predictor of the molecular alignment in an electric field. The epoxy analog of this molecule—MU12—has a large value of *Θ*, just like the other three molecules. Thus, we may expect negative dielectric anisotropy in the nematic phase of the investigated epoxy materials and perpendicular arrangement of the molecules with respect to an external electric field.

### 2.2. Electronic Structure and Stability

Frontier molecular orbital (FMO) calculations provide important parameters describing basic molecular properties, such as stability, optical and electronic properties, or charge transfer. The energy levels of the highest occupied molecular orbital (HOMO—donating electrons) and the lowest unoccupied molecular orbital (LUMO—accepting electrons) determine the width of the energy gap, which is somewhat equivalent to the band gap in inorganic materials. Large energy gap is related to insulating properties and high molecular stability. Small energy gap is characteristic of materials that are less stable but easily polarizable, which make them more attractive for the electronic industry.

**Figure 4:**
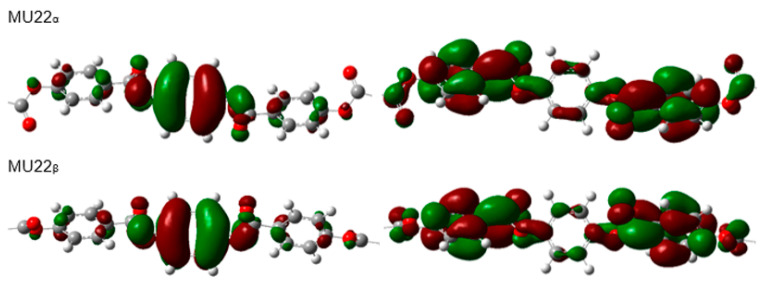

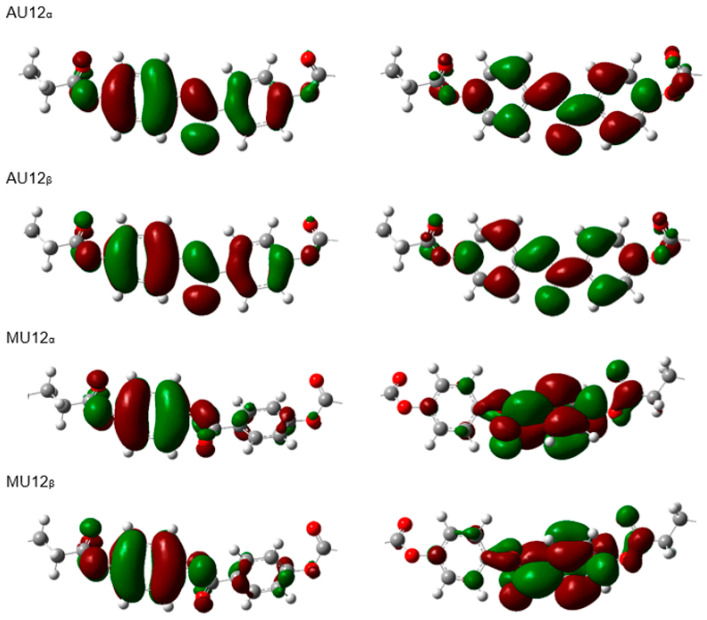
Visualization of frontier molecular orbitals—HOMO (**left**) and LUMO (**right**)—of the epoxy molecules: MU22, AU12 and MU12. Only the relevant, central parts of the molecules are shown.

In the case of the studied materials, we can compare the values of HOMO and LUMO energy and the energy gap for three different mesogens. The two different molecular conformations for each mesogen were selected for the calculations. Figure 4 visualizes central parts of the epoxy molecules: MU22, AU12, and MU12 with HOMO and LUMO isosurfaces (identical images were obtained for the vinyl analogs).

The terminal chains are excluded from the figure because only the central segment (which is identical in each epoxy and vinyl pair) participates in shaping the frontier orbitals. It can be noticed that both frontier orbitals have the same shape in both the more- and less-polar conformations, the only difference is in the inversed spatial arrangements of positive and negative isosurfaces of the orbitals (Figure 4).

Detailed results obtained for the epoxy materials and their vinyl analogs are compared in Table 4. The HOMO and LUMO energy values do not show any meaningful dependence on the mesogen polarization—these values are very similar for both selected conformations, in all the monomers. The energy gap width is not influenced by the polarization of terminal groups, either.

### 2.3. Non-Linear Properties

Non-linear optical (NLO) properties of materials play an important role in telecommunication and optoelectronic technologies on account of their applications in optical signal processing, electro-optical modulation for data storage, and harmonic generation or frequency mixing. Quantum chemical calculations enable obtaining values of linear polarizability (α) and first hyperpolarizability (β), which are very helpful in understanding the relation between the molecular structure and the NLO properties. Polarizability is a symmetric second-rank tensor with six independent coefficients (*α_xx_*, *α_xy_*, *α_yy_*, *α_xz_*, *α_yz_*, *α_zz_*)—coefficients with the same indices in different order have equal values. In the literature, the magnitude of average polarizability is often referenced:

(4) $$\langle \alpha \rangle =\frac{1}{3}\left(\alpha_{xx}+\alpha_{yy}+\alpha_{zz}\right)$$

The first hyperpolarizability is a third-rank tensor with 27 components, out of which 10 components are independent due to symmetry constraints (*β_xxx_*, *β_xxy_*, *β_xyy_*, *β_yyy_*, *β_xxz_*, *β_xyz_*, *β_yyz_*, *β_xzz_*, *β_yzz_*, *β_zzz_*). It is frequently replaced with the averaged magnitude calculated with the following formula:

(5) $$\beta_{tot}=\sqrt{{\left(\beta_{xxx}+\beta_{xyy}+\beta_{xzz}\right)}^{2}+{\left(\beta_{xxy}+\beta_{yyy}+\beta_{yzz}\right)}^{2}+{\left(\beta_{xxz}+\beta_{yyz}+\beta_{zzz}\right)}^{2}}$$

In isotropic materials with random packing of molecules, averaged values are usually a good reflection of the real strength of physical effects. In the case of liquid crystal materials, however, which exhibit macroscopic molecular order, the observed effects may have different strength in different directions. It is therefore important which components of polarizability and hyperpolarizability are non-zero. It is well visible in Table 5, which shows the polarizability components. In all the molecules, the largest component is *α_xx_*, indicating that the largest polarizability occurs in the direction of the principal molecular axis. Interestingly, its magnitude is very similar for polar and non-polar conformations of each molecule. Differences between bi- and tri-aromatic materials are also quite small. The mixed-index components of the polarizability tensor are all very small; only the diagonal components (in the coordinate system defined by the three main axes of the molecule) have significant values. In these materials, creating layers with enforced alignment will have large impact on the magnitude of the observed response.

Differences are also visible in the hyperpolarizability tensor components which contribute to the non-linear response in different directions (Table 6). Here as well, inducing bulk alignment of molecules may provide means of maximizing the material’s response.

In optoelectronic applications of organic or hybrid materials, creation of layers with specific internal order may be a key to their applicability. Similar conclusions may be drawn from the earlier mentioned study, whose authors noticed—while measuring electrooptical effect in liquid crystal materials—that only specific components of hyperpolarizability are of importance [1]. As mentioned in Introduction, nonlinear response in materials with rigid, bent mesogens measured along the *z* axis is determined only by *β_zxx_* and *β*_zzz_ [1]. In our case, the components of the first hyperpolarizability tensor are presented in Table 6. Inspection of these components shows that hyperpolarizability-related effects in these materials depend largely on direction—only selected components have significant values. Substantial differences between the polar and non-polar conformations of the same molecule are visible, as well as between bi- and tri-aromatic mesogens. In the case of tri-aromatic materials M22 and MU22, the only meaningful component is *β_zxx_*, and only in the conformations with nonzero dipole moment; hyperpolarizability of the centrosymmetric conformations is close to zero. Much larger values of hyperpolarizability components were found in bi-aromatic materials, with *β_xxx_* being the largest component and *β_zxx_* only slightly smaller. In this case too, differences exist between the less- and more-polar conformations—although polarity has little impact on *β_xxx_*, yet the component *β_zxx_* is larger by a factor of two in the polar conformation, in all the studied bi-aromatic molecules. An interesting property of AU12 compound is the significant magnitude of *β_xxy_* component, unlike the other materials. It is also noteworthy that the values of the hyperpolarizability tensor only depend on mesogens and are not affected by epoxy functional groups at ends of the chains. It means that the central, rigid mesogens are fully responsible for electrooptical properties of epoxy networks. To sum up, the values of hyperpolarizability components suggest that epoxy networks based on the studied monomers may show nonlinear response to an electric field applied in the direction of long molecular axes. The direction of the strongest nonlinearity depends on the length of the mesogen and the overall symmetry. In bi-aromatic mesogens it is the direction parallel to the long molecular axis (nonlinear effect determined by *β_xxx_*), and—to a somewhat lesser degree—perpendicular to it (components *β_xxy_* and *β_xxz_*). In tri-aromatic mesogens with non-centrosymmetric conformations the largest effect may be expected in the direction perpendicular to the main axis (component *β_xxz_*). Tri-aromatic conformations with central symmetry (M22_α_, MU22_α_) are not supposed to show nonlinear behavior, which is confirmed by low values of all the components of the obtained hyperpolarizabilities.

### 2.4. Orientation in the Electric Field—Experimental Results

**Figure 5:**
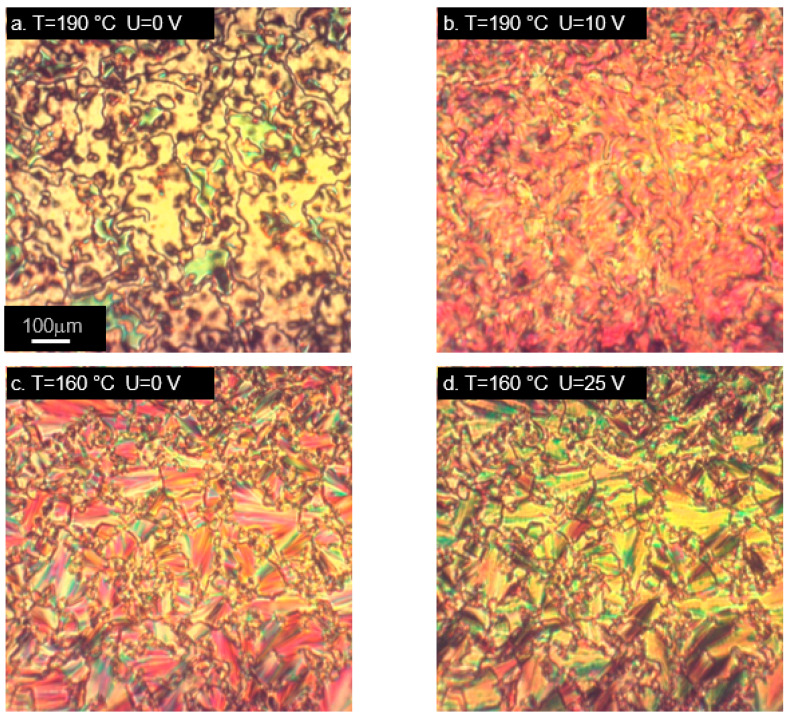

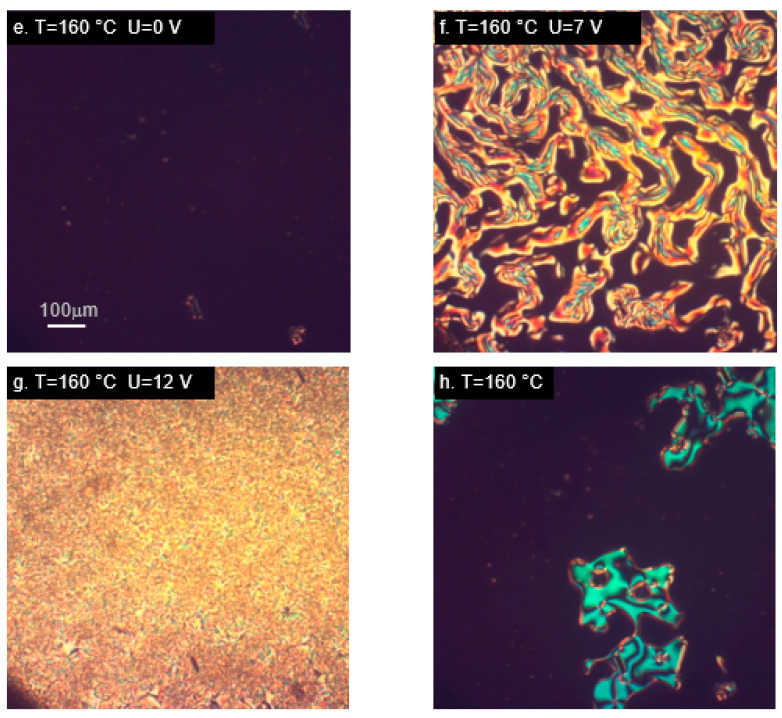
Microphotographs (in cross-polarized light) of tri-aromatic compounds M22 (**a**–**d**) and MU22 (**e**–**h**) in nematic (**a**,**b**,**e**–**h**) and smectic phase (**c**,**d**), showing alignment changes due to electric field. Samples placed in cells with layers inducing homeotropic orientation. (**h**) Vanishing of alignment after switching off the electric field.

In order to investigate the behavior of the studied materials under the influence of an electric field, we used a measurement cell which induces a homeotropic alignment of liquid crystal molecules. As mentioned earlier, the response to an electric field in liquid crystal phase is determined mainly by the value and direction of the dipole moment. In the studied molecules, its magnitude is very sensitive to the spatial conformation, but a large component perpendicular to the main molecular axis is present in all the cases. In tri-aromatic materials M22/MU22 a nematic phase appears at high temperatures, M22 also exhibits a smectic phase at lower temperatures. High temperatures of phase transitions are due to the molecular structure of the mesogen, these are also the temperatures used for curing epoxy materials with aromatic amines. The cell used for measurements induced a homeotropic alignment in all the cases except the M22 sample, whose alignment in the nematic phase was not as unequivocal, as in the other materials (Figure 5a).

M22 responded to the application of an electric field with texture changes (Figure 5b), but it was not the typical switching usually observed in nematic materials, due to the original lack of homeotropic alignment. An interesting behavior occurred in the smectic phase, which maintained its own texture, despite the alignment-inducing effect of the measurement cell (Figure 5c). A reaction to switching on the electric field was visible, but there was no change of the whole texture; only the molecules within domains were realigned (Figure 5d). This behavior was unlike any other studied case. Different result was obtained for the analogous material with terminal epoxy groups, MU22. In this case, behavior typical for nematics with negative dielectric anisotropy was observed (Figure 5e–h). Despite high temperature, the measurement cell was very effective in inducing the initial alignment of the samples. Changes dependent on the applied voltage were also clearly visible (Figure 5f,g), as well as restoration of the original alignment after switching off the electric field (Figure 5h).

**Figure 6:**
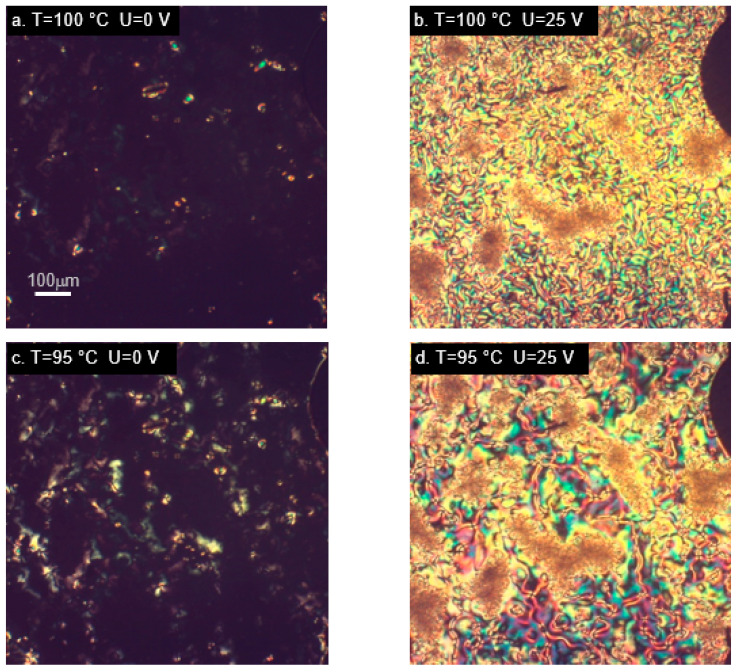
Microphotographs (in cross-polarized light) of compound AU12 in nematic (**a**,**b**) and smectic phase (**c**,**d**), showing alignment changes due to electric field. Sample placed in cell with omeotropically-ordering layer.

Interesting results were also observed in the epoxy material with two aromatic rings joined through an azoxy group—AU12. The initial homeotropic alignment was very good, both in the nematic and smectic phase (Figure 6a,c). After switching on the electric field, the material underwent substantial realignment like in materials with large transverse component of the dipole moment, indicating that the molecules adopted the orientation with main axes parallel to the electric field. AU12 was the only studied epoxy exhibiting a smectic phase. The observed smectic type A is characterized by low degree of order in comparison with other smectic types, which are difficult to realign. In the studied case, the smectic phase was as easily aligned by the measurement cell as the nematic phase (Figure 6c) and its alignment responded equally easily to the electric field (Figure 6d). Such a good realignment in reaction to the electric field may be due to the large value and the direction of the dipole moment of the investigated conformations. This behavior is different from one that was observed in the smectic phase of M22.

**Figure 7:**
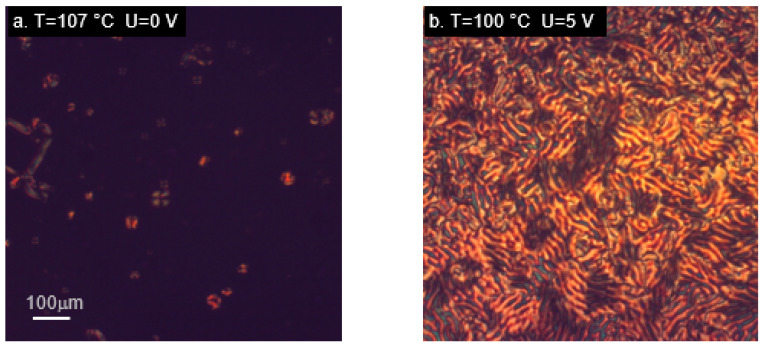

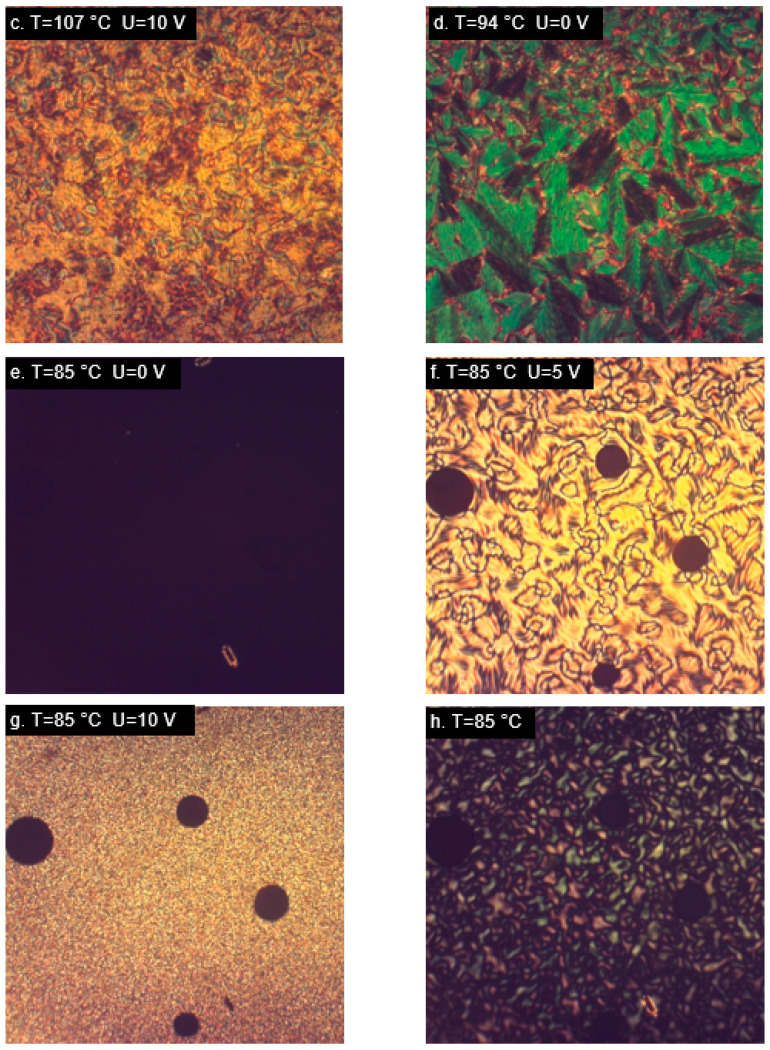
Microphotographs (in cross-polarized light) of compounds M12 (**a**–**d**) and MU12 (**e**–**h**) in nematic (**a**–**c**,**e**–**h**) and smectic phase (**d**), showing alignment changes due to electric field. Samples placed in cells with layers inducing homeotropic orientation. (**h**) Vanishing of alignment after switching off the electric field.

Observations of the materials with two aromatic rings joined through carboxyl groups (M12 and MU12) in the nematic phase yielded similar results (Figure 7).

The images obtained between crossed polarizers for cells with layers inducing a homeotropic alignment were almost uniformly black (Figure 7a,e), whereas switching on the electric field triggered visible realignment of the samples. In the case of M12, which has non-polar chains, textures observed in the nematic phase were different, depending on the applied voltage (Figure 7b,c); larger voltages made the microscopic image brighter, which agrees with the expectations. Small changes in the textures were also visible in the smectic phase of that material (Figure 7d), though these were only local changes rather than a complete change of the texture type. The epoxy material MU12 also underwent realignment in response to the electric field, with some changes in the texture depending on the applied voltage (Figure 7f,g). The original alignment was restored after switching off the voltage—the vanishing of the texture was captured in Figure 7h.

The conducted observations lead to a conclusion that epoxy materials are much easier to align both by preparing the cell surface and by applying an electric field. In all the cases, the molecules adopted the orientation with main axes perpendicular to the direction of the electric field. It is noteworthy that all the epoxy monomers displayed a homogeneous alignment in the measurement cell, better than the alignment of their vinyl analogs. Realignment of the samples due to the electric field also occurred more easily in epoxy materials, which mainly have a nematic phase. The observed behavior of the epoxy materials was in line with the expectation that an electric field should align the molecules perpendicularly to the direction of the field, due to the large component of the dipole moment perpendicular to the main molecular axis.

## 3. Materials and Methods

### 3.1. Materials

**Figure 8:**
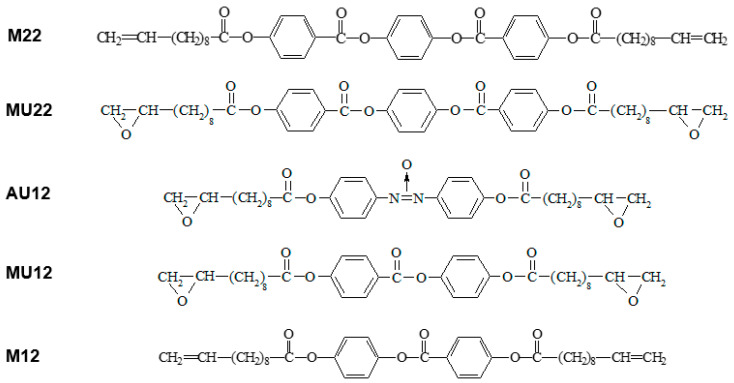
Molecular structure of the investigated compounds and symbols used instead of full chemical names.

The three investigated compounds have typical mesogenic cores with two or three aromatic rings linked by polar groups, terminated at both ends with aliphatic chains containing polar epoxy groups. The two analogous materials with non-polar vinyl groups were also studied to enable comparative analysis. The molecular structure of all the five materials is shown in Figure 8.

The synthesis path, temperatures of phase transitions and identification of liquid crystal phases were presented elsewhere [24,25,26]. A nematic phase was observed in all the studied materials, whereas the vinyl compounds also displayed a smectic phase. Temperatures of phase transitions are shown in Table 7. The appearance of a nematic phase in all the cases allows for controlling the molecular alignment also during curing, which makes it possible to compare experimental results with the behavior predicted on grounds of theoretical computations.

### 3.2. Analytic Methods

Textures of the compounds were observed in a polarizing microscope with crossed polarizers and recorded using a BIC-2S 1/2” CMOS digital camera (BIC Camera, Tokyo, Japan). The investigations were carried out using a cell made from two parallel glass plates covered with indium tin oxide (ITO) conducting layers and an extra layer enforcing homeotropic orientation of LC samples. The sample thickness in this cell was 5.5 µm and the sample area was about 25 mm^2^. A TMS 91 hot stage with an auxiliary electric field connector (Linkam, Epsom, UK) was used to conduct the observations. Conductivity measurements were performed using a circuit comprising an 8808A digital multimeter (Fluke, Eindhoven, The Netherlands) which works with currents above 1 nA and an SDP‑2603 DC (Manson, Hong Kong) power supply operating as a voltage source.

Ab initio calculations of molecular structure, dipole moments and first hyperpolarizabilities were performed using Gaussian 09 program suite [27] running on an institutional computing cluster. Molecular energies and wavefunctions were computed according to Density Functional Theory (DFT) model, using B3LYP hybrid functional [28,29,30,31,32] with standard basis set 6–31G*. Structural parameters of the studied molecules were determined by means of the Berny geometry optimization algorithm, based on an earlier algorithm published by H.B. Schlegel [33]. Correct execution of geometry optimization was verified by computing vibrational frequencies at the obtained stationary points: absence of imaginary values in the output confirms convergence to a true energy minimum rather than some transition state (saddle point). Dipole moments and hyperpolarizabilities were obtained from analytic derivatives of energy with respect to the applied electric field, using static frequencies. HOMO and LUMO surfaces were visualized in GaussView 03 program, using default rendering parameters.

## 4. Conclusions

Epoxy materials capable of forming composites with internal order resembling the molecular arrangement in liquid crystals are interesting due to the prospects of their applications in optoelectronics. Calculations performed on the studied molecules revealed large dipole moment components perpendicular to the main molecular axis, which give rise to negative dielectric anisotropy. Application of an electric field during curing of these compounds is therefore expected to produce polymer networks with specific internal order, where molecules are aligned perpendicularly to the direction of the applied field. This expectation is supported by the strong tendency of the studied molecules to acquire orientation perpendicular to the external electric field, experimentally observed in nematic phase.

Results of further calculations may serve as guidelines to maximize nonlinear response of ordered composites based on the studied mesogens. Analysis of hyperpolarizability components suggests that epoxy networks based on the studied monomers may produce nonlinear response when an electric field is applied along the mean direction of the principal molecular axes. The direction of the strongest NLO effect depends on the mesogen size and conformational symmetry. In materials with bi-aromatic mesogens the effect is stronger and mainly parallel to the main axis, while in tri-aromatic materials with non-centrosymmetric mesogen conformation it is only perpendicular to the main axis (NLO effect is not expected when inversion symmetry is present).

FMO calculations show the energy gap to be determined almost exclusively by the chemical structure of the mesogen, with very little influence of conformational changes. On the contrary, the values of the dipole moment and the first hyperpolarizability are very sensitive to mesogen conformation.

## Figures and Tables

**Table 1:** Results of geometry optimization for M22 and MU22. The angles δ_i_ describe the initial conformation. *E* is the B3LYP energy after optimization. Values of the dipole moment: *μ—*total, *μ*_M_—mesogen, *μ*_eo—_epoxy chain. *Θ* is the angle between the total dipole moment and the principal molecular axis. The two chosen conformations for each molecule are highlighted.

Initial Conformation	Optimized Structure				
δ_1_, δ_2_, δ_3_, δ_4_ (°)	*E*_B3LYP_ (Ha)	*μ* (D)		*Θ* (°)	Symbol
		*μ*_M_ (D)	*μ*_eo_ (D)		
**75, 120, 105, −120**	**−2232.41195087**	**0.3**		**88**	**M22** **_α_**
		**0.0**	**0.4**		
75, 120, 75, 120	−2232.41187634	0.3		90	M22
75, −120, 105, 120	−2232.41184511	0.4		78	M22
75, −120, −105, −120	−2232.41181277	0.1		90	M22
**75, −120, 75, −120**	**−2232.41170039**	**2.6**		**90**	**M22** **_β_**
		**2.2**	**0.4**		
75, 120, −105, 120	−2232.41165861	2.1		89	M22
75, 120, −75, −120	−2232.41149737	2.2		89	M22
75, −120, −75, 120	−2232.41147492	2.3		89	M22
**75, 120, 105, −120**	**−2382.81374195**	**0.7**		**89**	**MU22** **_α_**
		**0.0**	**2.1**		
75, 120, 75, 120	−2382.81366538	3.6		90	MU22
75, −120, 105, 120	−2382.81363725	1.0		86	MU22
75, −120, −105, −120	−2382.81359203	3.3		90	MU22
**75, −120, 75, −120**	**−2382.81348624**	**2.2**		**90**	**MU22** **_β_**
		**2.2**	**2.1**		
75, 120, −105, 120	−2382.81350061	2.3		88	MU22
75, 120, −75, −120	−2382.81329249	3.6		89	MU22
75, −120, −75, 120	−2382.81324045	3.2		88	MU22

**Table 2:** Results of geometry optimization for AU12. The angles δ_i_ describe the initial conformation. *E* is the B3LYP energy after optimization. Values of the dipole moment: *μ*—total, *μ*_m—_mesogen, *μ*_eo—_epoxy chain. *Θ* is the angle between the total dipole moment and the principal molecular axis. The two chosen conformations are highlighted.

Initial Conformation	Optimized Structures				
δ_1_, δ_2_ (°)	*E*_B3LYP_ (Ha)	*μ* (D)		*Θ* (°)	Symbol
		*μ*_m_ (D)	*μ*_eo_ (D)		
**60, −120**	**−1959.27603366**	**3.9**		**67**	**AU12_α_**
		**1.6**	**2.1**		
60, −60	−1959.27597586	1.3		50	AU12
		1.6	2.1		
**60, 120**	**−1959.27593243**	**5.1**		**73**	**AU12** **_β_**
		**2.5**	**2.1**		
60, 60	−1959.27593164	3.1		75	AU12
120, −120	−1959.27573355	2.3		25	AU12
120, 60	−1959.27565102	6.6		79	AU12
120, 120	−1959.27564991	4.7		66	AU12
120, −60	−1959.27559634	4.3		74	AU12

**Table 3:** Results of geometry optimization for M12 and MU12. The angles δ_i_ describe the initial conformation. *E* is the B3LYP energy after optimization. Values of the dipole moment: *μ*—total, *μ*_m_—mesogen, *μ*_eo_—epoxy chain. *Θ* is the angle between the total dipole moment and the principal molecular axis. The chosen conformations for each molecule are highlighted.

Initial Conformation	Optimized Structure				
δ_1_, δ_2_, δ_3_ (°)	E_B3LYP_ (Ha)	*μ* (D)		*Θ* (°)	Symbol
		*μ*_m_ (D)	*μ*_eo_ (D)		
**60, 120, 45**	**−1812.79456691**	**2.0**		**38**	**M12_α_**
		**2.0**	**0.4**		
60, 120, −45	−1812.79453283	2.0		37	M12
		1.9	0.4		
**60, −120, −45**	**−1812.79452681**	**2.9**		**51**	**M12_β_**
		**2.9**	**0.4**		
60, 60, −45	−1812.79450621	1.8		11	M12
60, 60, 45	−1812.79449519	2.8		52	M12
60, −120, 45	−1812.79448290	1.9		19	M12
60, −60, 45	−1812.79422750	2.9		46	M12
60, −60, −45	−1812.79417161	2.8		43	M12
**60, 120, 45**	**−1963.19638534**	**4.7**		**69**	**MU12_α_**
		**2.0**	**2.1**		
60, 120, −45	−1963.19638204	3.8		65	MU12
		1.9	2.1		
**60, −120, −45**	**−1963.19635377**	**5.5**		**70**	**MU12_β_**
		**2.9**	**2.1**		
60, 60, −45	−1963.19633532	2.3		35	MU12
60, −120, 45	−1963.19633065	2.7		47	MU12
60, 60, 45	−1963.19630662	5.0		67	MU12
60, −60, 45	−1963.19602995	2.7		32	MU12
60, −60, −45	−1963.19598906	4.2		57	MU12

**Table 4:** HOMO and LUMO energy and band-gap values for the selected conformations of the studied molecules.

Molecule	HOMO (Ha)	LUMO (Ha)	Band Gap (eV)	Dipole (D)
MU22_α_	−0.233	−0.056	4.82	0.7
MU22_β_	−0.234	−0.055	4.87	2.2
M22_α_	−0.232	−0.054	4.84	0.3
M22_β_	−0.234	−0.055	4.87	2.6
AU12_α_	−0.217	−0.079	3.76	3.9
AU12_β_	−0.217	−0.079	3.76	5.1
MU12_α_	−0.233	−0.054	4.87	4.7
MU12_β_	−0.232	−0.054	4.84	5.5
M12_α_	−0.232	−0.053	4.87	2.0
M12_β_	−0.231	−0.053	4.84	2.9

**Table 5:** Components of the polarizability tensor for the selected conformations of the studied molecules. Values are given in atomic units (1 a.u. = 1.649 × 10^−41^ C^2^⋅m^2^⋅J^−1^).

Molecule	*α_xx_*	*α_xy_*	*α_yy_*	*α_xz_*	*α_yz_*	*α_zz_*
MU22_α_	799	−14	412	−7	−8	294
MU22_β_	793	−26	359	0	0	349
M22_α_	812	−8	401	−6	−9	288
M22_β_	807	−21	343	0	0	346
AU12_α_	722	21	357	21	1	251
AU12_β_	720	−18	355	−1	−25	256
MU12_α_	621	1	345	−2	7	262
MU12_β_	622	−3	334	−3	−23	273
M12_α_	635	−1	328	−10	5	261
M12_β_	635	−6	320	6	−28	270

**Table 6:** Components of the hyperpolarizability tensor for the selected conformations of the studied molecules. The values are given in atomic units (1 a.u. = 3.206 × 10^−53^ C^3^⋅m^3^⋅J^−2^).

Molecule	*β_xxx_*	*β_xxy_*	*β_xyy_*	*β_yyy_*	*β_xxz_*	*β_xyz_*	*β_yyz_*	*β_xzz_*	*β_yzz_*	*β_zzz_*
MU22_α_	−4	7	7	7	0	−14	−9	−7	−12	28
MU22_β_	0	0	0	0	373	−132	30	0	0	−37
M22_α_	0	−3	−9	5	29	17	−6	7	−2	−11
M22_β_	0	0	0	0	408	−108	25	0	0	−53
AU12_α_	750	771	77	16	85	117	9	2	36	−2
AU12_β_	−753	741	−60	20	−267	23	−7	−15	27	51
MU12_α_	−664	382	60	40	−121	4	−4	−3	44	26
MU12_β_	660	−295	−54	−49	208	86	22	−69	−53	−41
M12_α_	−688	283	62	−1	−129	21	2	−3	−7	7
M12_β_	662	−223	−69	1	230	96	5	−57	−10	−1

**Table 7:** Phase transitions in the investigated compounds [5,24,25,26]. Cr, Cr1, Cr2—crystal phases; SmA, SmC, SmF—smectic phases; N— nematic phase; I—isotropic phase.

Compound	Temperatures of Phase Transitions (°C)	Route
M22	Cr_1_ → 130 (Cr_2_) 136 (SmC) → 168 (N) → 192 (I) Cr → 125 (SmC) → 166 (N) → 192 (I)	heating cooling
MU22	Cr_1_ → 78 (Cr_2_) → 138 (N) → 189 (I) Cr_1_ → 125 (N) → 185 (I)	heating cooling
AU12	Cr → 90 (SmA) → 97 (N) → 110.5 (I) Cr → 80 (SmA) → 97 (N) → 110 (I)	heating cooling
M12	Cr → 78 (SmA) → 90 (N) → 97 (I) Cr → 71 (SmF) → 74 (SmC) → 90 (N) → 97 (I)	heating cooling
MU12	Cr → 79 (N) → 89 (I) Cr → 67(N) → 90 (I)	heating cooling

## Data Availability

Data sharing is not applicable to this article.
